# The Scientific Basis of Homœopathy

**Published:** 1852-05

**Authors:** 


Art. VI.—*The Scientific basis of Homœopathy.* By William H. Holcombe, M. D. Cincinnati: H. W. Derby & Co. 1852: p. p. 295, 12mo.

This book professes not only to give "the scientific basis of Homœopathy," but to furnish a refutation of the argument of Dr. Hooker against that system, contained in the admirable little volume noticed in our last number. It is about twice the size of Dr. Hooker's volume and at least ten times as heavy. Dr. Hooker's is calm, dignified, fair, and logical; this is such a production as one would expect from a physician who had deserted the regular profession and turned homœopathist. It is not meant for the profession but the people. The author is evidently a disaffected practitioner, who hopes to make capital out of the desertion and abuse of his scientific brethren. His style is adapted to the classes to which his appeal is made; what he meant to be the "scientific basis" of his book, no one can understand; but it will not, on that account, be any the less acceptable to the genuine believer in homœopathy, whose motto is *credo quia impossibile est.* He thinks homœopathy must triumph over orthodox medicine, because this new sect devotes itself to the study of *nerve-force* instead of the liver, stomach and other coarse organs. The following extract contains an expression of his creed:

The Nerve Force is apparently of a much higher and more complicated character than any of the Physica Forces with which we have been comparing it. It may be called analogically, the *solar force vitalized.* It is the subtlest and most powerful form under which the One Universal Force manifests itself. But after all, it is to be remembered, that it is only the force of inorganic nature raised, like an algebraic expression, to a higher power. It is matter in motion, and as such, susceptible of conversion into other and more familiar forms of motion. It is resolvable into Heat, Light, Electricity, Magnetism, Chemical Action, and Mechanical power. Upon its modes and manifestations, therefore, all the phenomena of living bodies, in health or disease, depend.

A more distasteful work than the one before us has seldom fallen in our way, and we dismiss it with the pledge not soon again to touch the subject of which it treats. A good deal of experience and observation have satisfied us, that the good accomplished by the exposure of quackery is not great, and that we may safely leave it to run its course and die a natural death. A certain amount of it seems to be a natural ingredient of human society. It is continually changing but never disappears. One absurdity comes in the room of another, as by a process which chemists style displacement, but the mass neither increases nor grows less. And so, we suppose, it will continue, human nature remaining unchanged, "to the last syllable of recorded time."

